# Aquatic macroinvertebrates stabilize gravel bed sediment: A test using silk net-spinning caddisflies in semi-natural river channels

**DOI:** 10.1371/journal.pone.0209087

**Published:** 2019-01-02

**Authors:** Lindsey K. Albertson, Leonard S. Sklar, Scott D. Cooper, Bradley J. Cardinale

**Affiliations:** 1 Department of Ecology, Evolution, and Marine Biology, University of California-Santa Barbara, Santa Barbara, CA, United States of America; 2 Department of Ecology, Montana State University, Bozeman, MT, United States of America; 3 Department of Earth and Climate Sciences, San Francisco State University, San Francisco, CA, United States of America; 4 Department of Geography, Planning and Environment, Concordia University, De Maisonneuve Blvd. W. Montreal, Quebec, Canada; 5 School for Environment and Sustainability, University of Michigan, Ann Arbor, MI, United States of America; 6 Cooperative Institute of Great Lakes Research (CIGLR), University of Michigan, Ann Arbor, MI, United States of America; Brandenburgische Technische Universitat Cottbus-Senftenberg, GERMANY

## Abstract

Organisms can have large effects on the physical properties of the habitats where they live. For example, measurements in laboratory stream microcosms have shown that the presence of silk net-spinning insect larvae (Trichoptera: Hydropsychidae) can increase the shear force required to initiate movement of riverbed sediments. Few studies, however, have moved beyond laboratory settings to quantify the engineering impacts of aquatic insects under more complex field conditions. To bridge the gap between small-scale laboratory experiments and natural stream ecosystems, we conducted experiments in large (50 m^2^) outdoor river channels where net-spinning aquatic insects were manipulated in sediment patches that were 5 to 25 times larger than in previous studies. We tested whether larvae of two caddisfly species (*Arctopsyche californica* and *Ceratopsyche oslari*) influenced the stability of gravel during simulated floods when alone in monoculture and together in polyculture. On average, populations of caddisflies increased the critical shear stress required to initiate sediment movement by 20% compared to treatments without caddisflies. Per capita effects of caddisflies on sediment stability were similar between previous laboratory studies and this field experiment, and *Arctopsyche* had a larger per capita effect than *Ceratopsyche*, perhaps because of its larger size and stronger silk. Contrary to prior laboratory flume results, the effects of the two species on critical shear stress when together were similar to the additive expectation of both species when alone, but effects of the two species together were higher than the additive expectation when we accounted for density. Comparisons of total population and per capita effects suggest that caddisfly density, identity, and coexisting species likely have effects on the magnitude of caddisfly impacts on critical shear stress. Our findings imply that consideration of both the abundances and traits of ecosystem engineers is needed to describe and model their effects on sediment mobility.

## Introduction

The influence of organisms on their abiotic environment has been the focus of a long history of research [1–3]. Some of these biological impacts on physical processes are quite surprising, such as the degree to which jellyfish alter ocean mixing dynamics [4], microbes control snow precipitation patterns [5], or plant roots drive river meandering [6,7]. Organisms that create or modify physical habitats are commonly called ecosystem engineers [8,9]. In freshwater ecosystems, recent research has highlighted the importance of integrating ecosystem engineers into sediment transport models [10–12] and suggests that the magnitude of ecosystem engineer effects on sediment dynamics depend on organism identity, density, and biomass, as well as hydraulic energy and the physical characteristics of substrate [13,14].

Laboratory and microcosm experiments have been the primary tool used to isolate and measure biological engineering effects in streams [6,15–17]. Extrapolation of these results to natural systems is problematic, however, because of the limited spatial scale and simplification of stream habitats in these experimental settings, which may not capture the effects of sediment heterogeneity, flow conditions, community composition, and organism density under natural conditions. Impacts of a stream fish, the flannelmouth characin (*Prochilodus mariae*), on fine sediment accural in the field, for example, vary with the spatial heterogeneity associated with riffles versus pools and the presence of other fish species [18]. Field tests of the effects of freshwater ecosystem engineers on physical conditions have been rare owing to the difficulties of manipulating most taxa at large scales under complex flow conditions (but see [19,20]), but studies emphasize the value of comparing laboratory and field experiments, both to validate laboratory findings and to determine how, when, and to what degree measured effects persist at the field scale [21–24].

Several microcosm studies have demonstrated that net-spinning caddisfly larvae can stabilize sediments in flowing-water systems (Table 1). Hydropsychid caddisfly larvae (Trichoptera: Hydropsychidae) attach silk threads to riverbed substrate to anchor nets that they use to filter food from the water column. Caddisfly silk nets can alter current velocities and near-bed turbulence [25,26], as well as increase sediment stability by binding riverbed sediments together. These silk nets can increase the critical shear stress required to initiate sediment motion by as much as two-fold with consequences for streambed erosion over annual and decadal time scales [27–30]. Silk structures constructed by caddisfly larvae also have been shown to influence marlstone deposits and riverbed microtopography, preserving long-term records of flow direction in fossil deposits [31,32]. The results of laboratory experiments where caddisfly colonization, initial sediment motion measurements, or both have been conducted under flume conditions have been broadly consistent with the results of a single correlational field study indicating that caddisflies increase the force needed to mobilize streambed gravels [33]. Because the force required to initiate sediment motion affects bedload sediment flux, the size and spacing of bedforms, and ultimately river channel geometry, the effects of caddisflies on sediment dynamics could be fundamentally important to the physical and chemical characteristics of streams with repercussions for nutrient cycling, and habitat and water quality [34–36].

**Table 1:** Description of related studies.

Reference	Location of caddisfly colonization	Location of sediment movement estimates	Caddisfly species	Patch size (m^2^)	Caddisfly density (no./m^2^)	Surface grain size (mm)
This study	Semi-natural outdoor channels	Semi-natural outdoor channels	*Arctopsyche californica; Ceratopsyche oslari*	0.150	200–650	22
Albertson et al. 2014a	Laboratory	Laboratory microcosm	*Arctopsyche californica; Ceratopsyche oslari*	0.015	1,530–2,460	22
Albertson et al. 2014b	Laboratory	Laboratory microcosm	*Arctopsyche californica; Ceratopsyche oslari*	0.015	1,100–1,700	10, 22, 45, 65
Johnson et al. 2009	Field	Laboratory microcosm	*Hydropsyche angustipennis; Hydropsyche pellucidula; Hydropsyche contubernalis*	0.027	113–1,053	4–6, 6–8
Cardinale et al. 2004	Laboratory	Laboratory microcosm	*Hydropsyche depravata*	0.006	904–2,542	4
Statzner et al. 1999	Field	Laboratory microcosm	*Hydropsyche siltalai*	0.015	66–3,241	12–40

The range of experimental conditions used in studies that have investigated the stabilizing effects of caddisfly silk on streambed sediments.

Most previous work on caddisfly ecosystem engineering has focused on the sediment stabilizing effects of single caddisfly species; however, hydropsychid caddisflies are a diverse group, often with several species living in the same stream. Different species build silk nets that differ in architecture and tensile strength, suggesting that their roles as biogeomorphic agents will vary depending on which species are present [37,38]. In a previous laboratory experiment we found that combinations of caddisfly species had different effects on sediment stabilization than those predicted from each species' effect when alone (non-additive effects;[30,38]). Because caddisflies are territorial and compete for net-building sites [39], they often partition space within benthic sediments [40], which can lead to increases in gravel stabilization during floods when different species build nets at different sediment depths [30]. Owing to the limited spatial scale of previous laboratory experiments, non-additive effects of different caddisfly species on sediment stabilization have been observed only under standardized conditions in small flumes where caddisfly densities are high and individuals do not disperse [30,38]. Although the density-dependent biogeomorphic effects of engineer diversity have been documented for other macroinvertebrates, little is known about the potential density-dependent effects of caddisfly diversity on sediment stabilization [16]. The combined effects of the nets of different caddisfly species on sediment stability have not been experimentally examined in field settings, which limits extrapolation to natural conditions or across spatial scales [23,41,42].

This study represents a step toward bridging the gap between laboratory experiments and field experiments in natural settings. This experiment was conducted in large (1 m wide x 50 m long), outdoor, meandering semi-natural river channels with alternating pools and riffles, which were designed to resemble natural streams. Channels were underlain by substrate typical of Sierran streams and were fed by water from Convict Creek, providing water chemistry, suspended particle loads, physical characteristics, and periphytic and invertebrate communities similar to those in a natural stream [43,44]. Measurements of the shear stress needed to move bottom substrate with or without caddisflies present were conducted within these channels in small patches (0.15 m^2^) containing gravel with vertical size distributions and coarse armoring typical of gravel-bedded streams [45]. Measurements of initial sediment motion represent an important transition from a stable to a mobile bed that ultimately drive river sediment dynamics, including long-term sediment flux, the temporal distribution of transport, and channel geomorphology, such as the width and spacing of bars [35,46]. Our experimental protocols, then, created working patches necessary to measure sediment movement under different flow conditions while being embedded in semi-natural channels with physical, chemical, and suspended particle conditions resembling natural streams. Although the short colonization time of working patches (three days) probably resulted in biofilm, detrital, and invertebrate assemblages different from those in the surrounding channels, previous studies have reported that the shear stress needed to mobilize sediments did not differ between gravel substrate without hydropsychids that were and were not pre-conditioned for three to eight weeks in streams, indicating that these biological conditions did not affect sediment movement [27,29]. In contrast to previous studies on the shear stress required for sediment movement, we manipulated hydropsychids and measured sediment movement directly in our outdoor arenas and our working patches were five to 25 times larger than arenas used in previous studies. Finally, because most previous studies have examined the effects of single or amalgams of hydropsychid species on substrate movement [30,38], this study represents an advance in examining the individual and combined effects of different hydropsychid species on sediment mobilization under semi-natural conditions, allowing us to link the impacts of diversity and of different ecosystem engineers on physical processes. Although our results were still limited to patches and not to whole reach or stream network scales, our larger-scale manipulations allowed the microhabitat preferences of different hydropsychid species, alone and together, to influence gravel stabilization [25,47–49].

In summary, we addressed the following questions in this study: Are the effects of caddisflies on the critical shear stress required for sediment movement detectable in sediment patches an order of magnitude greater, and under more complex outdoor conditions, than those used in previous experiments? If so, are the effects of caddisflies on incipient sediment motion in more realistic, semi-natural stream channel patches similar in magnitude to the effects detected in the laboratory? Do species identity and interspecific interactions influence the engineering effects of caddisfly larvae on sediment movement under these semi-natural conditions? We hypothesized that caddisflies would increase the critical shear stress necessary for sediment movement, that the per capita effect of the larger-bodied *Arctopsyche* would be greater than that of the smaller *Ceratopsyche*, and that the effects of both species when together would be greater than the additive effects predicted from the effects of each species when alone; however, we expected the magnitude of these effects to be smaller in the semi-natural channels than in the laboratory because of the high caddisfly densities and restricted caddisfly dispersal typical of laboratory microcosm experiments [30,38].

## Methods

### Study organisms

Insects in the net-spinning caddisfly family Hydropsychidae are geographically widespread and diverse [50]. They are among the most abundant aquatic insects, reaching densities from hundreds to tens of thousands per square meter in fast-flowing streams [27,51,52]. In California’s Sierra Nevada where we conducted our experiment, *Arctopsyche californica* and *Ceratopsyche oslari* are especially abundant, reaching peak densities in late spring and early summer during and after spring snow-melt floods [53,54]. *Arctopsyche* has a body length twice and body mass seven times that of *Ceratopsyche* [30]. The hydropsychids (3^rd^ and 4^th^ instars) used in this experiment were collected from McGee Creek (latitude 37°35´N, longitude 118°47´W) and Convict Creek (37°36´N, 118°97´W), which are the streams closest to the outdoor experimental stream facility at the University of California’s Sierra Nevada Aquatic Research Laboratory (SNARL) near Mammoth Lakes, CA, USA (latitude 37°36´N, longitude 118°49´W). This study was performed in accordance with all University of California regulations.

### Experimental stream channels

**Fig 1:**
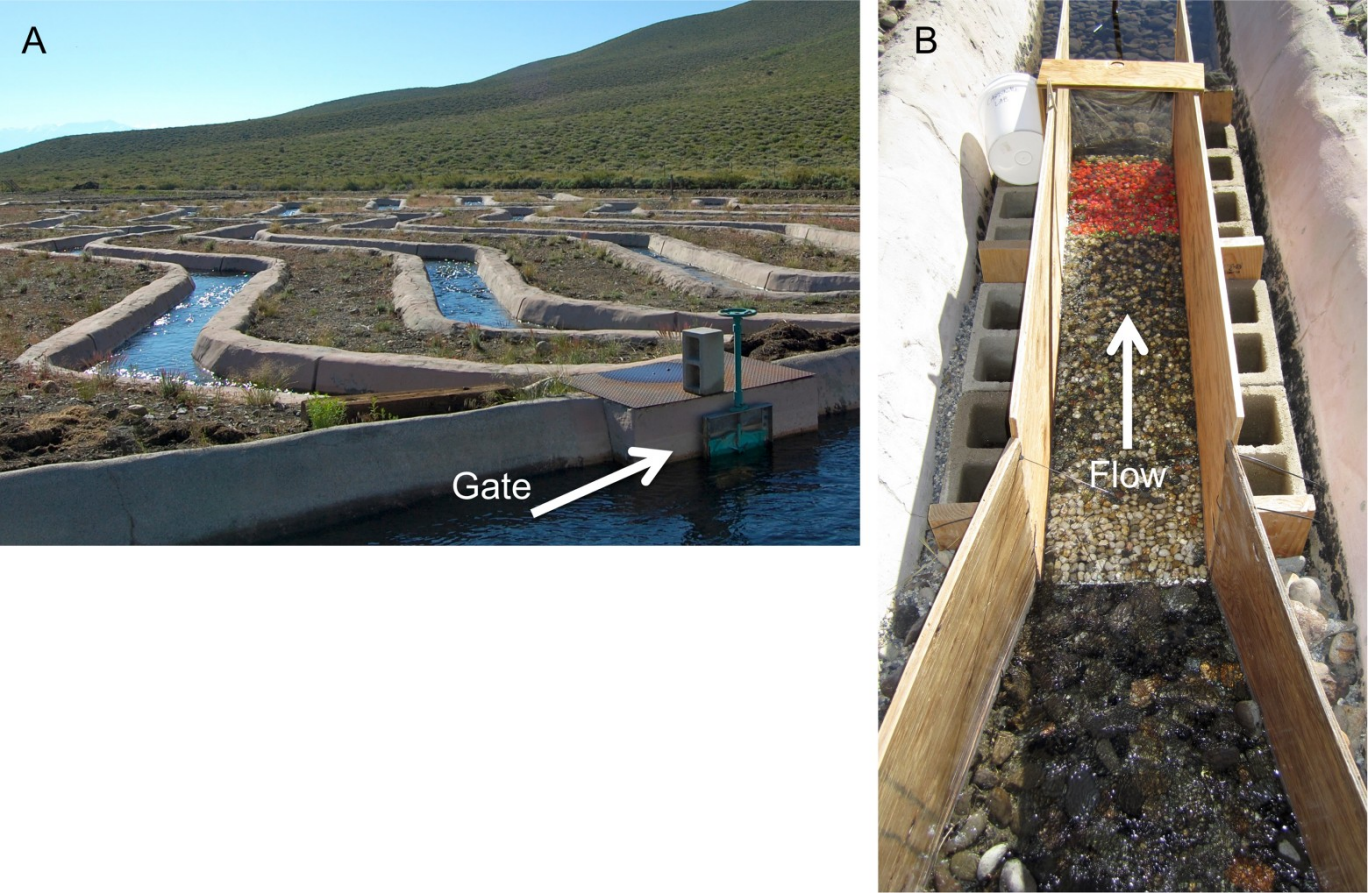
(A) The stream channels used in the experiment at the Sierra Nevada Aquatic Research Laboratory (SNARL), Mammoth Lakes, CA. Each of the four meandering channels is 50 m long by 1 m wide, and flow is controlled by a head gate (bottom right corner of the image for the first channel). (B) Plywood flumes used in the experiment. Sediments were glued to the plywood bottoms extending 1 m upstream from an experimental patch of moveable sediments (colored orange) where caddisflies built silk nets.

The experiment was conducted in four concrete-lined, meandering stream channels at SNARL that were each 50 m long by 1 m wide, containing alternating pools at meander bends and riffles in straight sections (Fig 1A; [43,44]). Water was delivered to each experimental channel via gravity flow through a feeder canal from a branch of adjacent Convict Creek, and channel depth and flow were controlled using adjustable gates located at the head of each channel. Flow was held at a uniform discharge until the simulated flood (see following section). Natural sand, gravel, and cobble substrate covered channel bottoms (D_16_ = 22.9, D_50_ = 31.6 mm, D_84_ = 41.7 mm).

At a straight riffle section in each of the four channels, we excavated the bed sediments and embedded a plywood flume (1.5 m long by 0.35 m wide), with bed slopes that varied from 1.2 to 1.9% (Fig 1B). The flumes were narrower than the concrete channel to increase flow depth and resulting bed shear stress. The flume entrance walls were angled to gradually converge flow toward the experimental sediment patches (0.46 m long by 0.35 m wide), which began 1 m downstream of the flume entrance. The upstream and downstream ends of the experimental sediment patches were vertically flush with surrounding substrates of the same size, which were glued to the plywood so that water flowed over stable sediment with similar roughness as it approached the experimental patches. Moveable sediments in the working patch were sorted vertically by hand to create a coarse surface layer (median grain size (*D*_*50*_*)* = 22 mm) and fine subsurface layer (*D*_*50*_ = 10 mm), typical of gravel-bedded rivers [45]. The patches were constructed by placing a subsurface layer that was two grain-diameters thick (total of 20 mm) in the flume, then covering the subsurface grains by hand with a layer of surface grains one-diameter thick (total of 22 mm). With this flume slope, sufficient shear stress was generated to mobilize bed sediment when the channel gates were opened to simulate a flood. The design of the sediment patches in this experiment were chosen to be similar to our previous work in the laboratory [30]. Average grain size of the patch surface in both experiments was 22 mm, underlain by a finer subsurface layer.

### Experimental design and caddisfly colonization

The experiment was designed as a randomized complete block in which the four caddisfly treatments (control with no hydropsychids, *Ceratopsyche* alone, *Arctopsyche* alone, both *Ceratopsyche* and *Arctopsyche* together) were randomly assigned to each of the four experimental channels at each time. The experiment was then replicated in nine temporal blocks in the summers of 2010 and 2012 (extending from July 11 to August 4, 2010, and from May 15 to June 26, 2012). Although we did not quantify invertebrates that drifted into experimental sediment patches during the experiment, we kick-sampled invertebrate assemblages in the surrounding channels and found that the assemblage was dominated by mayfly (Baetidae, Heptageniidae) and riffle beetle (Elmidae) larvae, with hydropsychids being very rare, agreeing with previous research in the SNARL channels that reported natural biofilm and invertebrate communities [44]. Because the flumes were open to the channel habitat, this experiment allowed caddisflies to interact with the surrounding invertebrate community, such as mobile taxa immigrating into the sediment patches.

We supplemented caddisfly densities in each experimental sediment patch by stocking patches with additional larvae collected from Convict and McGee Creeks. Caddisflies were carefully moved by hand from gravels and cobbles on the riverbed bottom into buckets with oxygen bubblers, where they were held at ambient stream temperature until they were added to experimental patches. To achieve our target caddisfly density of 2,000/m^2^, which was similar to densities found in nearby streams and used in previous laboratory experiments [55, 30], each 0.15 m^2^ experimental patch was stocked with 300 hydropsychid larvae at the beginning of each temporal block, with 150 *Arctopsyche* and 150 *Ceratopsyche* added to each polyculture patch. Caddisfly larvae were placed in the water just upstream of the sediment patch and allowed to drift onto experimental substrate. Larvae were given three days to settle and construct silk nets. During the first 24 hours of each temporal block, any individuals drifting past the experimental sediment patch into a downstream net (1 mm mesh) were manually reintroduced to the upstream end of the sediment patch. After 24 hours, drifting caddisflies were captured and retained in the downstream net for the remaining two days of the colonization period. We did not routinely quantify the number of drifters into the nets.

Although we set a target caddisfly density at the initiation of each experimental block, individuals were often lost to predation or emigration resulting in a density lower than the target [30]. As a consequence, we used two methods to calculate caddisfly densities in the experimental patches at the time of the simulated flood (see following section). In the first year, any caddisflies that were dislodged, either during a given flow stage or after all remaining sediment had moved into the sediment trap at the end of each simulated flood, were captured in a 1 m long, 1 mm^2^ mesh net just downstream from the sediment trap, then enumerated. Caddisfly density in a patch was calculated as the sum of all caddisfly individuals captured across all flow stage increments and at the end of each flood, divided by the sediment patch size (0.15 m^2^). In the second year, we did not have enough personnel to simultaneously control the gates, watch for sediment motion, and count caddisfly larvae, so we created sacrificial sediment patches before the start of the 2012 temporal blocks that allowed us to estimate patch caddisfly density by counting nets visible at the bed surface, similar to Statzner et al. 1999. Nets were easily visible and identifiable by their characteristic shapes and colors, and the same researcher counted nets across all channels and temporal blocks. The sacrificial patches had a gravel size distribution and arrangement identical to the experimental patches and hydropsychids were added as for the experimental patches. After the three day colonization period, we counted the number of visible nets in four patches of each monoculture treatment and three patches of the polyculture treatment, then thoroughly disturbed patch sediments by hand, counted all caddisflies collected in the downstream net, and correlated the number of observed caddisfly nets with the number of caddisfly individuals collected in each patch for each species treatment (R^2^ = 0.91 and R^2^ = 0.87 for *Ceratopsyche* and *Arctopsyche* monocultures, respectively). In relating the number of observed nets to benthic densities of each species in polyculture, we assumed a ratio of 60 *Ceratopsyche* to 40 *Arctopsyche* for the polycultures based on relative densities measured in the 2010 experiments. During the flood experiments in 2012, we counted the number of visible nets in each patch before each flood, then calculated benthic densities using the relationships described above.

### Simulated flood

After the hydropsychid colonization period, we simulated a high discharge event in the channels to determine the shear stress required to initiate sediment motion in patches assigned to each caddisfly treatment. We gradually increased flow by slowly opening the head gate for each channel (Fig 1A) until we visually observed the movement of at least one surface gravel into a sediment trap located 8 cm downstream from the experimental patch. The same researcher made these observations for each channel and each temporal block. The sediment trap was a plastic, rectangular box (61 cm long, 15 cm wide, and 15 cm deep) that was embedded in the bottom sediments and vertically flush with the downstream end of the plywood flume. When sediments were first observed to move, discharge was held constant for three minutes and any transported grains were collected from the sediment trap. The gate was then opened incrementally to increase the discharge at three-minute intervals until all or most of the sediment in the experimental patch had moved into the sediment trap.

At each flow stage we measured water depth with a meter stick at the upstream entrance to each experimental patch. With measured values of flow depth and bed slope we calculated bed shear stress, *τ*_*b*_, for each flow increment as *τ_b_* = *ρ_w_ghs* where *ρ*_*w*_ is the fluid density, *g* is the acceleration due to gravity, *h* is the depth of water, and *s* is the bed slope [55,56]. To estimate the critical shear stress (τ_crit_), or the threshold force per unit area required to induce sediment motion, we weighed the sediments collected in the trap for each incremental increase of flow and calculated the percentage of sediment particles moved at each shear stress. We then fit a log-normal curve to the percentage of particles moved as a function of shear stress and used this relationship to estimate the shear stress when 1% of the surface grains had moved [46]. We chose the reference erosion rate of 1% of the sediment mass moved because it estimates the stress close to the threshold of sediment motion while also considering chance vagaries in the first grain moved (e.g., the first grain to move might be located in an unstable location or not be anchored by a caddisfly net) [46].

### Data analysis

Because of differences in environmental conditions (e.g., flows, bed slopes) between years and among individual channels, we observed consistent differences in critical shear stress values across years and channels. We adjusted critical shear stress values for individual treatment replicates for year and channel effects by adding residual values for individual replicates from a general linear ANOVA model relating critical shear stress to channel, year, and channel*year interactions to the overall mean critical shear stress value. These adjusted values were then used in data presentations and statistical analyses of treatment effects (S1 Table). Expected values of critical shear stress for the polycultures were calculated as the monoculture values for each temporal block weighted by the density of each hydropsychid species in the associated polyculture. In addition to the critical shear stress response to treatments, we calculated the log response ratio (LRR) for caddisfly effects on sediment movement to estimate the proportionate increase above control values in critical shear stress caused by caddisfly treatments (hereafter, total LRR) as $total\;LRR=ln\left(\frac{\tau_{crit\;treatment}}{\tau_{crit\;control}}\right)$. To determine whether differences between this study and a previous laboratory study [30] might be explained by differences in caddisfly density, we also calculated the LRR per hydropsychid (hereafter, per capita LRR) by dividing total LRR by the measured (2010) or calculated (2012) caddisfly density in each replicate. A one-way ANOVA was used to compare total LRRs and per capita LRRs between this study and Albertson et al. [30] for all caddisfly treatments, and one-tailed paired t-tests were used to compare total LRRs and per capita LRRs between all pairs of treatments within each study (treatment values paired by temporal block). Following the diagnostics for log response ratios outlined in Hedges et al. 1999 [57], our response values met parametric assumptions. We used one-way ANOVA to test for differences in hydropsychid densities between the two years across all caddisfly treatments. All analyses were performed using R 2.14.1 [58] or JMP version 11 [59].

### Survey of natural caddisfly densities

To examine the implications of our experimental results for potential ecosystem engineering in natural streams, we compared hydropsychid densities in our experiment to those measured in four gravel-bedded creeks near our study site. Caddisflies were sampled from reaches of McGee, Convict, Rush, and Swauger Creeks, which drain the eastern side of the Sierra Nevada near the SNARL experimental channels. We removed sediments by hand from one square meter of streambed in each of three riffles in each creek on June 20, July 11, and July 26, 2012, and then counted the number of caddisfly nets in these sediments. Although we could not identify caddisfly individuals to genus or species using these methods, we did obtain estimates of total hydropsychid net density across these four streams. Values reported are the means (and SEs) of the three riffles across the three sampling dates for each creek.

## Results

**Fig 2:**
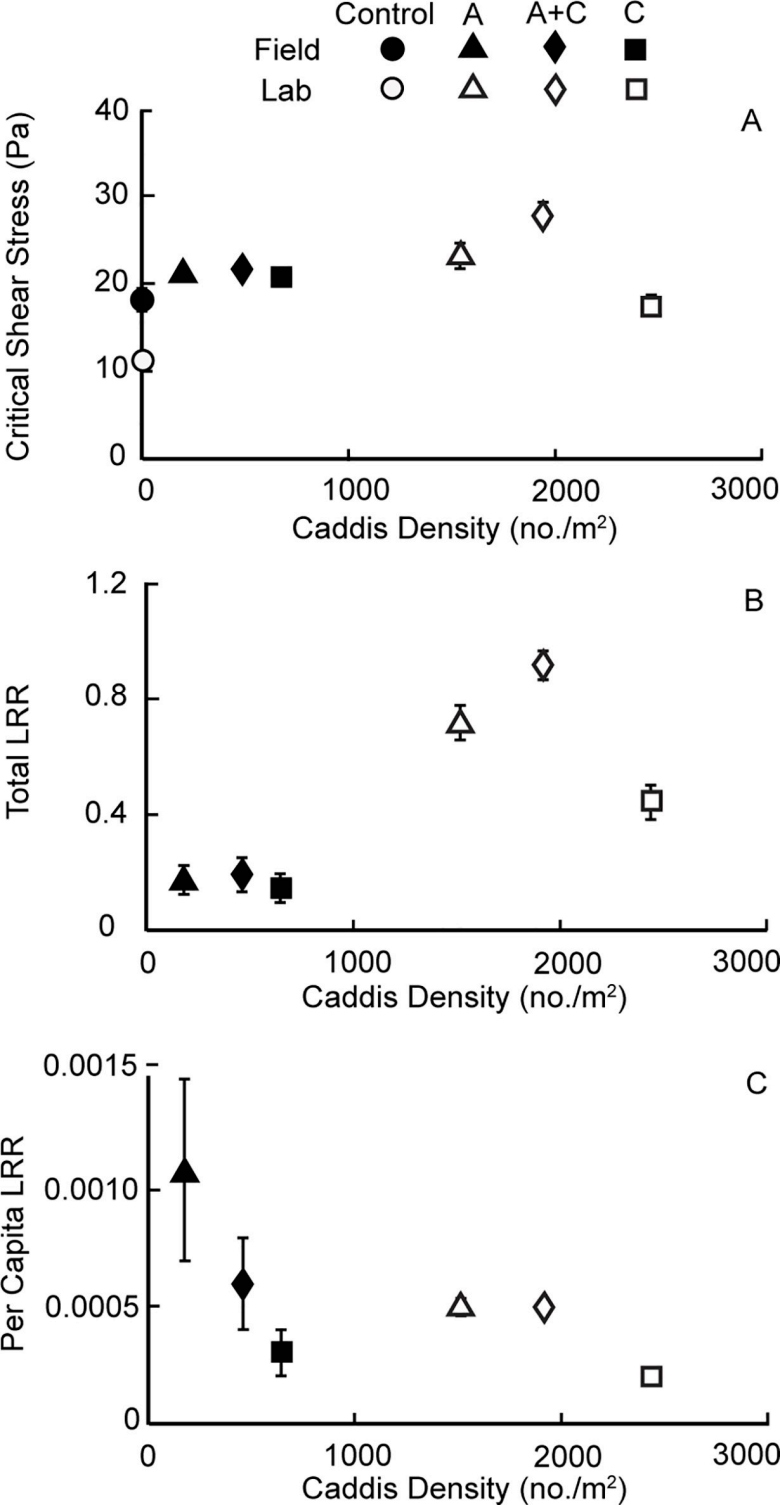
(A) The critical shear stress required to initiate sediment motion (in Pascals ± 1 SEM, equivalent to average force per unit area in Newtons/m^2^), measured across the average caddisfly densities (no./m^2^) found in the four experimental treatments in both the semi-natural channels (solid symbols, this study) and a previous laboratory study (open symbols, Albertson et al. 2014a). The figure includes data from control treatments without caddisflies (Control, circles) and experimental treatments containing *Arctopsyche californica* alone (A, triangles), *Ceratopsyche oslari* alone (C, squares), and both species together (polyculture, A + C, diamonds). (B) Total log response ratio (LRR) values across average caddisfly densities in different caddisfly treatments in the semi-natural and laboratory experiments. (C) Per capita LRR values across average caddisfly densities in different caddisfly treatments in the semi-natural and laboratory experiments. Where error bars are not visible for a given data point, they are subsumed within the symbol.

In this semi-natural experiment, we found that critical shear stress was significantly higher in all caddisfly larvae treatments than it was in controls without caddisflies (one-tailed paired t-tests, *n* = 7–9: *t*'s = 2.1–3.2, all *P*'s < 0.05; Fig 2A, Table 2). Although this result was qualitatively consistent with previous laboratory experiments, differences in critical shear stress among caddisfly treatments, and between observed and expected values of critical shear stress for polycultures, were not consistent between the semi-natural and laboratory experiments. In the experiment under semi-natural conditions, sediment stabilization tended to be greater, albeit not significantly greater, in the polyculture treatment than in the *Ceratopsyche* monoculture treatment (one-tailed paired t-test: *t* = 1.8, *P* = 0.08), but the effects of the *Arctopsyche* treatment on sediment stability were similar to those for the polyculture and *Ceratopsyche* treatments (one-tailed paired t-tests: *t* = 0.9, *P* = 0.27 and *t* = -0.1, *P* = 0.51). Critical shear stress in the polyculture treatment was similar to the additive polyculture expectation based on critical shear stress values for the monocultures (one-tailed paired t-test: *t* = 1.3, *P* = 0.11).

**Table 2:** Results from the experiment.

Comparisons of main treatment effects				
Treatment	Density (No./m^2^)	Critical shear stress (Pa)^Ŧ^	LRR	per capita LRR
Control	0	18.1±1.1a		
*Arctopsyche*	180±30	21.1±0.6b	0.17±0.05a	0.0011±0.0004b
*Ceratopsyche*	650±80	20.7±0.4b	0.14±0.06a	0.0003±0.0001a
Polyculture	470±60	21.7±0.8b	0.20±0.05a	0.0006±0.0002b
Comparisons of diversity effects				
Treatment	Density (No./m^2^)	Critical shear stress (Pa)^Ŧ^	LRR	per capita LRR
Polyculture (observed)	470±60	21.7±0.8a	0.20±0.05a	0.0006±0.0002b
Polyculture (expected)	20.8±0.5a	0.15±0.06a	0.0003±0.0002a

Values with the same letter within the same column are not significantly different (p > 0.05, paired one-tailed t-test). Values are means ± 1 SE for *n* = 7 to 9. Caddisfly density estimates are rounded to the nearest 10. The LRR represents log response ratio and per capita LRRs were estimated by dividing the LRR by the caddisfly density for each replicate.

^Ŧ^ reported values are standardized across years and channels

By definition, critical shear stress and total LRR values across caddisfly treatments showed similar patterns (Fig 2A and 2B), but total LRR values in the semi-natural channels were much lower than those found in the laboratory (one-way ANOVA, study effect: F_1,46_ = 75.5, *P* < 0.001; Fig 2B). This finding can be explained, in part, by differences in caddisfly density, which were higher in the laboratory than in the semi-natural channels. Standardizing effect sizes by hydropsychid density showed that per capita effect sizes for a given treatment were similar in the semi-natural and laboratory experiments (ANOVA, study effect: F_1,46_ = 2.2, *P* = 0.14) (Fig 2C). In both the semi-natural and laboratory experiments, per capita LRRs were not significantly different in the *Arctopsyche* and polyculture treatment; however, values for both of these treatments were significantly greater than those for the *Ceratopsyche* treatment (one-tailed paired t-tests, *P*’s ≤ 0.05, Fig 2, Table 2). In addition, observed values for polyculture per capita LRR in both the semi-natural channels (one-tailed paired t-test, *P* = 0.005, t = 3.7) and the laboratory (one-tailed paired t-test, *P* = 0.003, t = 3.9) were greater than additive expectations (Fig 2, Table 2).

**Fig 3:**
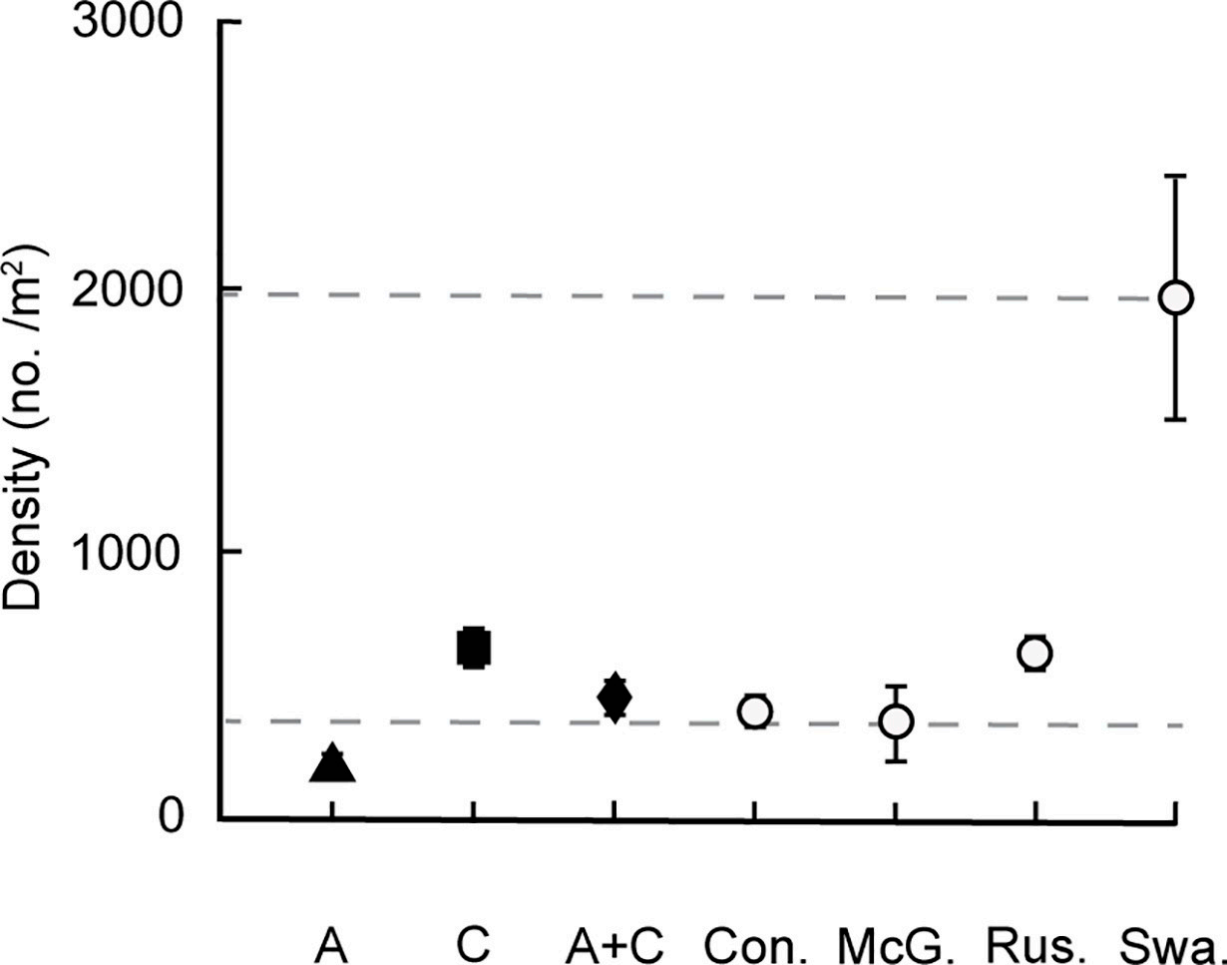
Caddisfly densities in the semi-natural experiment (solid symbols) for *Arctopsyche* monoculture (A), *Ceratopsyche* monoculture (C), and polyculture (A + C) treatments, and total caddisfly silk net densities in four streams (open symbols), including Convict (Con.), McGee (McG.), Rush (Rus.), and Swauger (Swa.) Creeks, near the SNARL field station in Mammoth Lakes, CA, USA. Values are means ± 1 SEM, and dashed lines represent the minimum and maximum mean larval caddisfly densities measured over three dates in each of the four streams.

Caddisfly densities in the semi-natural experiment were notably lower than target densities, and substantially lower than those used in previous laboratory experiments (Fig 2). The densities of caddisflies in the semi-natural experimental patches, when each species was alone, averaged 180 ± 30 individuals/m^2^ for *Arctopsyche* and 650 ± 80 individuals/m^2^ for *Ceratopsyche* (Fig 3). The polyculture treatments averaged 460 ± 50 individuals/m^2^ with a 60:40 ratio of *Ceratopsyche* to *Arctopsyche* densities (280 *Ceratopsyche* and 180 *Arctopsyche*/m^2^). Measured final caddisfly densities represented 10–33% of the individuals originally introduced to each patch, perhaps due to high drift rates or mortality. Caddisfly density was different between the two years of the experiment (ANOVA: F_3,32_ = 21.7; *P* < 0.001) and across caddisfly treatments (F_3,32_ = 37.5; *P* < 0.001), but there was no significant interaction effect between year and caddisfly treatment (F_3,32_ = 0.5, *P* = 0.64), indicating that caddisfly densities were higher across all treatments in 2012 than in 2010.

Average caddisfly silk net density in four streams near the experimental study site was 840 nets/m^2^, ranging from 360/m^2^ in McGee Creek to 1,960/m^2^ in Swauger Creek (Fig 3). Caddisfly densities in our semi-natural experiment (average = 425/m^2^ in monoculture, 460/m^2^ in polyculture) were within the range of densities measured in natural streams but generally lower than those used in other experimental manipulations investigating the impact of caddisflies on sediment stability (Table 1). Further, high caddisfly densities in one stream (Swauger Creek) were similar to those used in our previous laboratory experiments, indicating that caddisfly densities in past laboratory experiments approximate those found in some streams.

## Discussion

A growing body of literature has advanced our understanding of the role that animal ecosystem engineers play in river sediment dynamics, but the few studies that have moved beyond controlled microcosm experiments to larger-scale field experiments have revealed unexpected or incongruent results [18,60]. In this study, we used an experiment in semi-natural channels and patches to demonstrate that an abundant and ubiquitous group of aquatic macroinvertebrates can influence the force required to initiate sediment movement. We expected that various factors in the semi-natural channels might influence how hydropsychids modify incipient sediment motion, including increased dispersal away from potential predators or competitors, settling at more natural and perhaps lower densities, adjustment of silk net architecture and silk quantity in response to variation in flow and increased food delivery, more heterogeneous substrate arrangements, or the overriding effects of floods on sediment mobility. We found that caddisfly nets increased critical shear stresses by an average of 1.2 X over the control compared to 1.6 to 2.5 fold increases in sediment stability noted in previous experiments [29,30]. Although the impacts of silk net-spinning hydropsychid populations on sediment motion were reduced in the outdoor arenas compared to previous laboratory experiments, the significant increase in sediment stability produced by hydropsychid silk nets in the present study, where densities were low compared to those found in many natural gravel-bedded streams and in previous microcosm experiments, provides further support for the conclusion that increases in critical shear stress due to caddisfly nets occur commonly in gravel-bed rivers and should be considered in models of sediment mobilization [14]. Furthermore, critical shear stress for hydropsychid populations in the laboratory showed the pattern polyculture > *Arctopsyche* > *Ceratopsyche*, and observed critical shear stress values exceeded additive expected values for polyculture [30]; however, these patterns were not observed in the semi-natural channels, perhaps because differences among species treatments depended on hydropsychid density. This conjecture was supported by the observation that patterns in per capita hydropscyhid effects were similar in the experiments conducted in the semi-natural channels versus the laboratory suggesting that hydropsychid densities would be a good predictor of their total population impacts on sediment stability, consistent with results reported in Statzner et al. (1999) [27].

We initially introduced caddisfly larvae to experimental sediment patches at a density of 2,000/m^2^, but final caddisfly densities averaged 425/m^2^. The low proportions (10–30%) of introduced individuals that settled and established on experimental substrate could be due to mortality or to emigration induced by either aggressive interactions among caddisfly individuals or by continued searching, via drift or crawling, for suitable net attachment space and preferred rock sizes, rock arrangements, and flow and food delivery patterns [30,48,61]. Previous studies have documented intense fighting between caddisfly individuals, which are known to aggressively defend their silk nets and kill or exclude competitors [39]. Such antagonistic behavioral interactions might regulate the capability of caddisfly populations to stabilize sediments by ultimately limiting caddisfly densities achieved in natural streams [62].

Our previous laboratory study suggested that interactions among caddisflies in polycultures resulted in the vertical partitioning of the sediment pore space, leading to non-additive increases in critical sheer stress [30]. However, in this semi-natural experiment we found little support for non-additive total effects of different species, when together, on sediment stabilization. The laboratory flumes used in our previous experiments continually recirculated water and reintroduced caddisfly larvae to experimental sediment patches, resulting in caddisfly densities of up to 2,500/m^2^, which may have forced more intense interactions between species than found in natural systems where individuals can drift or crawl out of patches to evade interactions or search for unoccupied substrate. These findings lead us to speculate that non-additive polyculture effects on physical processes in streams may depend on engineer densities [16], because the polyculture effects on critical shear stress that we detected in the laboratory [30] occurred at hydropsychid densities that were six times higher than those observed in the semi-natural experiment. Furthermore, per capita effects of *Arctopscyhe* and *Ceratopsyche* on sediment stability, when together, exceeded additive expectations, suggesting that interactions between these species drove their per capita effects in polyculture, effects that were not evident at the population level when densities were very low. Different net-spinning caddisfly species often coexist in nature, leading to the partitioning of resources and distribution patterns showing areas of overlap and areas dominated by a single species [47,49,63–65]. The influence of caddisfly species interactions on physical processes may be strongest in streams or local patches where caddisfly densities are high, such as those observed in Swauger Creek, but more evidence is needed to determine if multi-species assemblages of freshwater macroinvertebrates at a variety of densities have non-additive effects on physical processes similar to the non-additive effects of multiple species that have been observed in other systems [66,67].

We hypothesized that knowledge of species traits might improve the accuracy of our predictions of their biogeomorphic effects [16,30,68]. Despite relatively low densities, *Arctopsyche* alone increased the critical shear stress above control levels and the per capita effects of *Arctopsyche* were larger than those of *Ceratopsyche* in both semi-natural and laboratory experiments. These findings suggest that the total abundance or total biomass of hydropsychid populations may not always determine their engineering effects, particularly in multi-species assemblages [27]. *Arctopsyche* is larger and more aggressive than *Ceratopsyche* [30, 39], and builds larger and stronger nets [38], which may affect both its interactions with *Ceratopsyche* and its relative effects on sediment stability. Per capita caddisfly effects on sediment stability were similar in *Arctopsyche* and polyculture treatments, which both were higher than those in *Ceratopsyche* treatments, suggesting a dominant influence of *Arctopsyche* on sediment stabilization in polycultures. In general, caddisfly net characteristics, such as their shape, size, silk thread tensile strength, and number of silk threads per net, vary across species and environmental gradients, such as the size of substrate, food availability, and local current velocities, with probable repercussions for their roles as sediment stabilizers [40,69,70].

Because of their connections to a natural creek, the semi-natural channels in this experiment probably allowed for more realistic hydropsychid densities, food supplies, interactions with surrounding biological communities, and hydrochemical conditions compared to previous studies where caddisflies were manipulated in the laboratory [30,71] while still allowing us to estimate critical shear stress at larger spatial scales [72]. Although other previous studies allowed hydropsychids to colonize expermimental arenas in the field, then measured the effects of hydropsychids on shear stress in lab flumes, our study manipulated hydropscyhid densities, examined the individual and combined effects of two species, and measured critical shear stress in semi-natural, outdoor arenas with natural substrate, flow, food supply, and weather conditions. Nevertheless, the geomorphological and hydrodynamic conditions in the plywood flumes embedded within the stream channels did not completely replicate natural stream channels, which have greater heterogeneity in substrate and flow characteristics. We noted differences in control critical shear stress values in the laboratory versus our semi-natural channels, even though the different experiments used similarly-sized gravels, suggesting that scaling hydrodynamic processes between the two settings is challenging. Although the literature indicates that biofilm contributes to the stability of only sediments that are much finer than those we used and there were almost no hydropsychids present in our control patches, we cannot completely rule out the possibility that engineering organisms may have colonized and influenced the stability of the controls in this semi-natural experiment [73]. Although we report the stabilizing effects of caddisflies on a single grain size, caddisfly engineering impacts decrease with increasing grain size, so future work will need to address how these findings translate to streams with different and/or heterogeneous substrate conditions [38]. The relative importance of hydropsychid effects far exceeded the effects of physical conditions (spatial distribution and orientation of gravels) on sediment mobility in another study [27], but these biotic versus abiotic relative effects likely vary across time or space with different physical characteristics. The next step toward linking caddisfly larvae and sediment motion in entire streams should include experiments that either remove or add caddisfly larvae to whole stream reaches using, for example, experimental techniques such as exclosures or electricity, while allowing the accurate measurement of sediment movement under different flow regimes [19,60].

Our finding that the bed stabilizing effects of caddisfly larvae persist under more realistic semi-natural conditions has important implications for the linkages between ecosystem engineers and aquatic ecosystem dynamics. Sediment movement induced by high flows can increase the mortality and drift of stream animals, affect nutrient fluxes, and alter water quality. Future work should address how the modification of habitat by caddisflies influences other taxa, community composition, and food availability [74,75]. Both physical and biological factors driving sediment mobility need to be considered in predicting sediment loads under hydrologic regimes affected by global climate and land use changes [76–78]. We have used a case study with caddisfly larvae to show that common aquatic animals can reduce sediment mobilization, a potentially important consideration for developing predictive transport models that inform the design of restoration projects and generate knowledge about the evolution of fluvial landscapes [79,80]. Further, our results indicate that species identity and densities may influence biophysical interactions. Future progress on biophysical coupling in streams will require greater knowledge of the abundances, distributions, diversity, and traits of engineering species and how these characteristics relate to spatial and temporal heterogeneity in flow conditions, grain sizes, and thresholds of sediment motion [81,82].

## Supporting information

S1 Table Data repository. (XLSX)
